# Searching for New Genetic Biomarkers of Axial Spondyloarthritis

**DOI:** 10.3390/jcm11102912

**Published:** 2022-05-20

**Authors:** Bartosz Bugaj, Joanna Wielińska, Katarzyna Bogunia-Kubik, Jerzy Świerkot

**Affiliations:** 1 Department of Rheumatology and Internal Medicine, Wroclaw Medical University, Borowska 213, 50-556 Wroclaw, Poland; jerzy.swierkot@umw.edu.pl; 2 Laboratory of Clinical Immunogenetics and Pharmacogenetics, Hirszfeld Institute of Immunology and Experimental Therapy, Polish Academy of Sciences, R. Weigla 12, 53-114 Wroclaw, Poland; joanna.wielinska@hirszfeld.pl (J.W.); katarzyna.bogunia-kubik@hirszfeld.pl (K.B.-K.)

**Keywords:** spondyloarthritis, ankylosing spondylitis, SNP, genetic biomarker, extra-articular manifestations, uveitis, treatment effectiveness, biologics

## Abstract

Background: Axial spondyloarthritis (axSpA) is a chronic inflammatory condition of the spine. In addition to musculoskeletal symptoms, there are also extra-articular manifestations. The aim of this study was to search for new biomarkers associated with the clinical presentation and treatment response in axSpA patients. Methods: In this study, 106 axSpA patients and 110 healthy controls were enrolled. Six single-nucleotide polymorphisms (SNPs) were selected for genotyping: *ERAP1* rs2287987, *ERAP2* rs2549782, *TNF* rs1800629, *TNFRSF1A* rs767455, *TNFRSF1B* rs1061622, and *FCGR2A* rs1801274. Participants were examined at baseline and after 12 and 24 weeks of anti-TNF therapy. Results: SNPs associated with high axSpA initial activity were *TNFRSF1A* rs767455 and *TNFRSF1B* rs1061622 (*p* < 0.008). The *ERAP1* rs2287987 *AA* genotype was more frequently observed in patients with enthesitis (*AA* vs. *G+*, *p* = 0.049), while the *TNFRSF1B* rs1061622 *GG* genotype was more common in participants with uveitis (*GG* vs. *TT*, *p* = 0.042). Potential in predicting anti-TNF treatment response was demonstrated by *ERAP1* rs2287987, *ERAP2* rs2549782, *TNFRSF1B* rs1061622, and *FCGR2A* rs1801274. Conclusions: SNPs can be used to identify patients at risk of severe disease to initiate treatment earlier. Genetic testing will allow clinicians to choose the right drug for the patient.

## 1. Introduction

Axial spondyloarthritis (axSpA) is a chronic inflammatory condition of the axial skeleton that can lead to chronic pain, structural damage, disability, and a loss of quality of life. axSpA can present with or without peripheral arthritis. The introduction of MR in diagnostics has made it possible to detect patients with early disease before the establishment of structural damage. axSpA encompasses both radiographic axSpA, in which patients have definite structural damage on radiographic assessment, and nonradiographic axSpA (nr-axSpA), in which patients typically have symptoms of chronic (often inflammatory) back pain with evidence of sacroiliitis on MRI in the absence of definite X-ray structural changes. Some patients with nr-axSpA may progress to r-axSpA, while others will never experience such progression [1]. In addition to musculoskeletal symptoms, there are also extra-articular manifestations, of which uveitis is the most common, with a prevalence of 23% in ankylosing spondylitis (AS) and 16% in nr-axSpA. Other extra-articular manifestations, such as psoriasis and IBD, are less common, occurring in 10% and 4% of AS patients, respectively. The incidence in nr-axSpA is similar [2].

Despite new methods, the average time to a correct diagnosis of AS has been estimated to be approximately eight years, with some estimates as high as fourteen years. Delayed diagnosis results in delayed treatment, which is related to higher disease activity, poorer quality of life, greater radiographic damage, and increased economic burden [3]. The large heterogeneity of patients, including in severity, long-term outcomes, and extra-articular symptoms, has also increased the awareness of the need for biomarkers to help predict clinical outcomes. Currently used biomarkers, such as HLA-B27 status, C-reactive protein (CRP), and erythrocyte sedimentation rate (ESR), have, at best, moderate diagnostic and predictive value. Inflammatory markers, such as calprotectin, have been investigated, but standardization is lacking, and they mirror CRP. Markers of bone metabolism, such as matrix metalloproteinase (MMP) and dickkopf-1 (Dkk-1), have shown diverging results on disease activity and progression. The polygenic risk score was shown to predict disease risk more accurately than HLA-B27 alone [4,5]. In addition, genetic biomarkers may provide an opportunity to identify patients at higher risk of severe disease.

Several questions arise about axSpA treatment. The current licensed drugs are nonsteroidal anti-inflammatory drugs (NSAIDs) used in the first-line treatment, and biologic disease-modifying antirheumatic drugs (bDMARDs) targeting tumor necrosis factor (TNF)-α or interleukin (IL)-17A, which are used in the following lines: Janus kinase (JAK) inhibitors are another promising therapeutic modality for axSpA. There is a lack of guidance on which biologic drug to use in a particular patient [6]. Further relevant questions concern whether drug treatment can be reduced in patients responding well, especially those in remission [7]. A question of whether a combination of an NSAID with a TNF-blocking agent is better than a TNF-blocking agent alone still awaits an answer in a prospective trial. We do not have data to help us decide which biologic or targeted synthetic DMARD to start or whether specific patients would be better candidates for one or the other DMARD. Similarly, we do not know the optimal sequence of switching between biologics or whether a combination of biologics (or a biologic and a targeted synthetic DMARD) might be an option in patients who have failed one or two biologics [8]. It is estimated NSAIDs may be ineffective in over 40% of patients, some of whom have to use reduced doses partly due to intolerance [9]. Ineffectiveness is also a problem with biologic drugs. A meta-analysis found that the drug survival for all TNF inhibitors was approximately 0.76 at year 1 and gradually decreased to approximately 0.51 at year 5 [10]. There is a need to find biomarkers that will make it easier to match the drug to the patient based on the prognosis of effectiveness.

The triggers and pathogenesis of axSpA are not yet completely understood. Many factors are involved. Due to external factors such, as mechanical stress or gut microbiome disturbances, genetic susceptibility amplifies multiple inflammatory innate and acquired immune responses, eventually leading to musculoskeletal damage and repair [11]. Two pathways in the inflammatory responses have attracted much interest—the TNF-α axis and the IL-23/IL-17 axis [12]. AS, the prototypical form of axSpA, is highly heritable. To date, apart from HLA-B27, over 100 non-MHC loci have been identified, contributing to 28% of the genetic variation in the disease [13].

One of genetic variants that was found to be associated with susceptibility to AS is *ERAP1* rs2287987 was firstly described in the genome-wide association study performed on British individuals (OR = 0.75, *p* < 0.001) [14]. The association between these polymorphisms and AS was observed in Spanish [15], Portuguese [16], Hungarian [17], Polish [18], and Iranian [19] populations. Meta-analysis studies also linked the minor alleles of the rs2287987 with AS development in European patients (OR = 0.708, 95% CI = 0.658–0.762, *p* < 1.0 × 10^−9^) [20]. Another meta-analysis showed that rs2287987 seems to be associated with AS in Caucasians (OR = 0.643, 95% CI = 0.543–0.762, *p* < 0.001) and overall populations (OR = 0.650, 95% CI = 0.559–0.756, *p* < 0.001) but not in Asians [21]. The molecular model of ERAP1 suggests a three-domain protein structure surrounding a central Zn atom. The rs2287987 (Met349Val) is located close to the catalytic center and affects enzyme activity [22].

In contrast to the *ERAP1* polymorphism, *ERAP2* rs2549782 is not associated with AS risk [23]. However, the amino acid variation leads to activity changes as well as specificity of the enzyme and thus influences the ability to cooperate with ERAP1 in antigen presentation [24].

A recent publication described the potential role of the rs1800629 variant located within *TNF-α* gene with an increased AS risk in Caucasians [25]. TNF-α receptors polymorphism, namely *TNFRSF1A* rs767455 and *TNFRSF1B* rs1061622, are associated with susceptibility, too [26].

Although polymorphisms associated with the risk of the disease may not influence severity, response to treatment, or clinical features, they constitute an important factor of immune response variability in humans. The above-mentioned studies prompt us to investigate those single-nucleotide polymorphisms (SNPs) in the homogeneous Polish population. Therefore, the aim of this study was to search for biomarkers associated with the clinical presentation and response to treatment of axSpA patients.

## 2. Materials and Methods

### 2.1. Study Group

The current study enrolled 106 patients with axSpA. All of them were Caucasians and were recruited from the Department of Rheumatology and Internal Medicine at Wrocław Medical University, Poland. The study group consisted of AS patients diagnosed according to modified New York criteria [27] and patients diagnosed with nr-axSpA using ASAS criteria [28]. All of them gave their informed consent to participate in the study. Clinical data such as age, body mass index (BMI), disease onset, HLA-B27 presence, peripheral arthritis history, and medication history were collected. The most commonly used DMARD was methotrexate (MTX), usually in patients with peripheral disease. Among them, some also used corticosteroids, mainly methylprednisolone at doses less than 10 mg converted to prednisone. Moreover, the following extra-articular manifestations were described: uveitis; enthesitis; psoriasis; and inflammatory bowel disease (IBD), defined as Crohn’s disease or ulcerative colitis. Patient characteristics are shown in Table 1. The exclusion criteria were underage, cancer, pregnancy or breastfeeding, chronic disease or organ failure exacerbation, mental retardation, and alcohol or drug abuse. The observation was started at the beginning of anti-TNF treatment after two NSAIDs were found to be ineffective or when contraindications to such treatment were present. Laboratory parameters such as CRP and ESR were measured. Disease activity was determined using the Bath Ankylosing Spondylitis Disease Activity Index (BASDAI) (range 0–10) [29], and back pain was quantified using the Visual Analogue Scale (VAS) (range 0–100 mm). The initial axSpA activity was high (BASDAI > 4). Patients received one of the following anti-TNF drugs: adalimumab, etanercept, certolizumab, golimumab, and infliximab. Participants were examined at baseline and after 12 and 24 weeks of therapy.

The control group consisted of 110 healthy individuals from the Regional Centre of Transfusion Medicine and Blood Bank in Wroclaw. Participants in the control group, without a history of rheumatic diseases, were matched to participants in the patient group in terms of age and sex.

Our research obtained permission from the Wroclaw Medical University Ethics Committee.

### 2.2. SNP Selection and Genotyping

Tested genetic variants were selected based on previous publications analysis and search results from NCBI Database of Short Genetic Variations (dbSNP) and SNPinfo Web Server [30]. Minor allele frequency (MAF) in EUR was above 10% (1000 Genomes Project) [31].

We selected six single nucleotide polymorphisms (SNPs) for genetic analysis: *ERAP1* rs2287987, *ERAP2* rs2549782, *TNF* rs1800629, *TNFRSF1A* rs767455, *TNFRSF1B* rs1061622, and *FCGR2A* rs1801274.

*ERAP1* rs2287987, *ERAP2* rs2549782, *TNFRSF1B* rs1061622, and *FCGR2A* rs1801274 polymorphisms are within protein-coding regions that can cause amino acid change (non-synonymous coding SNPs). In addition, *TNFRSF1B* rs1061622, *ERAP1* rs2287987, *ERAP2* rs2549782, and *TNFRSF1A* rs767455 are located at two base pair of intron-exon junction region, while exonic splicing enhancer (ESE) or exonic splicing silencer (ESS) may disrupt splicing activity and cause alternative splicing. Additionally, *TNF* rs1800629 location is predicted as transcription factor binding site (TFBS). Table 2 shows the SNPs’ characteristics.

Whole-blood samples were collected into ethylenediaminetetraacetic acid (EDTA) anticoagulant BD Vacutainer^®^ tubes (Becton, Dickinson and Company, Franklin Lakes, NJ, USA). Genomic DNA was extracted from blood using a QIAmp DNA Blood Kit (Qiagen, Hilden, Germany) according to the manufacturer‘s protocols. Genotyping was performed employing LightSNiP assays (TIB MOLBIOL, Berlin, Germany) on the LightCycler 480 II Real-Time PCR Instrument (Roche Diagnostics, Basel, Switzerland). From amplification and detection with specific probes by melting curve analysis, it is possible to obtain visual discrimination of normal and variant alleles in the homozygous and heterozygous status [32]. Genotyping was conducted according to the manufacturer‘s instructions.

**Table 2:** Characteristics of selected SNPs.

Gene	SNP ID	Allele	Location	Variant	1000 Genomes Allele Frequencies (EUR)	Function of Encoded Protein
ERAP1	rs2287987	A>G (T>C)	Chr5, exon 6	missense variant M (Met) > V (Val)	A(T): 0.775 G(C): 0.225	Trimming peptides before their binding to MHC class I molecules. ERAP1 and ERAP2 can physically interact forming a heterodimer. They are IFNγ- and TNFα-inducible enzymes. ERAP1 preferentially cleaves peptides with C-terminal amino acids, whereas ERAP2 presents a marked preference for N-terminal and shorter peptides.
ERAP2	rs2549782	G>T	Chr5, exon 7	Missense variant K (Lys) > N (Asn)	G: 0.480 T: 0.520	
TNF alpha G-308A	rs1800629	G>A	Chr6, promoter	upstream gene variant	G: 0.866 A: 0.134	Proinflammatory cytokine that plays a crucial role in inflammatory and immune diseases.
TNFRSF1A	rs767455	T>C	Chr12, exon 1	synonymous variant	T: 0.571 C: 0.429	Receptors for the TNF cytokine. TNFRSF1A is a dominant receptor involved in inflammatory and innate immune responses. TNFRSF1B mediates anti-inflammatory and homeostatic functions of TNF.
TNFRSF1B	rs1061622	T>G	Chr1, exon 6	missense variant M (Met) > R (Arg)	T: 0.782 G: 0.218	
FCGR2A	rs1801274	A>G	Chr1, exon 4	missense variant H (His) > R (Arg)	A: 0.489 G: 0.511	Removing antigen-antibody complexes in the circulation and transduction activating signals into cells via immune receptor when ligated with immune complexes. FCGR2A activate immune cell functions, including phagocytosis and the release of inflammatory mediators.

Based on: [33,34,35,36].

### 2.3. Statistical Analysis

The distribution of continuous variables was tested for normality by the Shapiro–Wilk test. The results are presented as medians with interquartile ranges for nonnormally distributed data or as numbers with percentages for categorical variables. The chi-square or Fisher‘s exact test was applied to compare genotypes and allele frequencies between the patient and control groups. The nonparametric Kruskal–Wallis test followed by an unpaired two-sample Wilcoxon test was performed to identify associations between genetic variants and clinical parameters. To analyze relationships between categorical data (e.g., treatment response) and genotypes, Fisher’s exact test was used.

Hardy–Weinberg equilibrium (HWE) was tested using an online calculator (Michael H. Court (2005–2008)).

A *p*-value lower than 0.05 (*p* < 0.05) was considered statistically significant. Bonferroni correction was used to adjust the significance of the *p*-value after the Mann–Whitney test.

Statistical analysis was performed using R software version 4.0.3 (http://www.r-project.org, accessed on 7 April 2022) and GraphPad Prism 7 for Windows.

## 3. Results

### 3.1. Genotype Distribution

The genotype distributions of the examined polymorphisms were in Hardy–Weinberg equilibrium (*p*-value range for controls: 0.11–0.72 and patients: 0.26–0.79). The frequency of genotypes and alleles did not differ between patients and healthy controls (Table 3). Data in Table 3 are presented as the number and percentage of individuals carrying a given genotype. The numbers and frequencies of alleles are also shown. It is worth noting that for the *TNF* rs1800629 polymorphism, the *p*-value was 0.06, which may indicate some trend between this polymorphism and the occurrence of axSpA. A more detailed analysis of men in the group showed that the *TNF* rs1800629 *GG* genotype was more common in patients than in controls (*GG* vs. *GA + AA*, *p* = 0.020, OR = 2.71, 95% CI 1.25–5.76).

### 3.2. Analysis of Potential Associations with Clinical Parameters

Initial analysis showed the following statistically significant associations between clinical parameters and polymorphisms: (i) *ERAP1* rs2287987 and CRP levels before and after twelve and twenty-four weeks of anti-TNF treatment; (ii) *TNFRSF1B* rs1061622 and BASDAI and VAS values before treatment; (iii) *TNFRSF1A* rs767455 and CRP and ESR levels before treatment; and (iv) *FCGR2A* rs1801274 and ESR levels after twenty-four weeks of therapy.

For the ERAP1 polymorphism, higher initial CRP values were observed among *AA* homozygotes (*AA* vs. *AG + GG*, *p* = 0.012) than among patients with the *G* allele. *AG* heterozygotes presented lower median CRP levels before therapy (*AA + GG* vs. *AG*, *p* = 0.009; *AA* vs. *AG*, *p* = 0.010) and after three (*AA + GG* vs. *AG*, *p* = 0.018; *AA* vs. *AG*, *p* = 0.025) and six months of anti-TNF treatment (*AA + GG* vs. *AG*, *p* = 0.009; *AA* vs. *AG*, *p* = 0.014; *AG* vs. *GG*, *p* = 0.048).

An analysis of the rs1061622 polymorphism within the *TNFRSF1B* gene showed that *GG* homozygotes were associated with higher VAS values (*TT + TG* vs. *GG*, *p* = 0.001; *TG* vs. *GG*, *p* = 0.001; *TT* vs. *GG*, *p* = 0.002) and BASDAI (*TT + TG* vs. *GG*, *p* = 0.005; *TG* vs. *GG*, *p* = 0.004; *TT* vs. *GG*, *p* = 0.009) before treatment than *T* allele carriers, either heterozygotes or *TT* homozygotes.

The genetic variants of the second TNF receptor gene, *TNFRSF1A* rs767455, also influenced the disease course. In the dominant (*TT* vs. *TC + CC*) and codominant (*TT* vs. *TC*) models, *TT* homozygotes had higher levels of CRP (*p* = 0.009 and *p* = 0.006) and ESR (*p* = 0.013 and *p* = 0.010) before therapy.

In a recessive model of the *FCGR2A* rs1801274 gene, ESR levels were lower in homozygotes than in carriers of the A allele (*AA + AG* vs. *GG*, *p* = 0.030) after six months of therapy. Investigating associations between described polymorphisms and clinical parameters are shown in Supplementary Material—Table S1.

However, after Bonferroni correction, only the associations described herein that linked the *TNFRSF1B* rs1061622 polymorphism with VAS and BASDAI parameters at baseline, as well as *TNFRSF1A* rs767455 with CRP levels before treatment, remained statistically significant (*p* < 0.008), and they are presented in Table 4.

### 3.3. Extra-Articular Manifestations and Form of the Disease

The occurrence of extra-articular manifestations in relation to genotype frequencies was analyzed. We observed that patients with the ERAP1 rs2287987 AA genotype more frequently presented with enthesitis (AA vs. G+, *p* = 0.049, OR = 4.636, 95% CI 1.101–21.24). The GG genotype of TNFRSF1B rs1061622 was more common in patients with uveitis than the TT genotype (GG vs. TT, *p* = 0.042, OR = 5, 95% CI 1.08–20). The same genetic variants in both polymorphisms were associated with higher CRP and VAS values. There were no significant relationships between the polymorphisms and IBD.

We also found that the *ERAP1* rs2287987 *A* allele (*AG + AA* vs. *GG*, *p* = 0.037, OR = 17.4, 95% CI 1.79–253; *AA* vs. *GG*, *p* = 0.026, OR = 24, 95% CI 2.23–349) and the *TNFRSF1B* rs1061622 *TT* homozygotes (*TT* vs. *GG*, *p* = 0.032, OR = 8.25, 95% CI 1.62–42.3) were more common among patients with HLA-B27 positivity. Other genetic associations with the presence of HLA-B27 were the more frequent appearance of the *ERAP2* rs2549782 *T* allele (*T +* vs. *GG*, *p* = 0.035, OR = 3.89, 95% CI 1.01–13) and the *GT* genotype (*GT* vs. *GG + TT*, *p* = 0.032, OR = 5.23, 95% CI 1.28–24.6; *GT* vs. *GG*, *p* = 0.015, OR = 7.5, 95% CI 1.60–38).

The disease was divided into two forms, namely axial and axial-peripheral, according to the involvement of spinal joints only or spinal and peripheral joints. The axial form was more common in *TNFRSF1A* rs767455 heterozygotes (*TC* vs. *TT + CC*, *p* = 0.0498, OR = 2.296, 95% CI 1.05–4.95). It is worth paying attention to this genotype. As mentioned above, *TC* carriers also had lower CRP and ESR levels before treatment. Furthermore, the only statistically significant relationship between medication used and genotypes was less frequent steroid intake in patients with the *C* allele (*TC + CC* vs. *TT*, *p* = 0.003, OR = 0.1901, 95% CI 0.061–0.54) and the *TC* genotype, as described in the codominant (*TC* vs. *TT*, *p* = 0.003, OR = 0.143, 95% CI 0.040–0.57) and overdominant (*TC* vs. *TT + CC*, *p* = 0.018, OR = 0.219, 95% CI 0.064–0.74) models.

### 3.4. Anti-TNF Treatment Effectiveness

The analysis of disease activity after three (3 mo) and six months (6 mo) of anti-TNF treatment assessed therapy outcome concerning the investigated genotypes. An absolute BASDAI score lower than three (<3) and two (<2) as well as disease activity reductions of more than 50% (>50%) and 75% (>75%) were evaluated. Values below 2 are defined as low disease activity and between 2–4 as moderate [37]. In other countries such as Spain, a value of ≤2 is taken as inactive disease and <4 as low activity [38]. According to the criteria of the Polish National Health Fund, we adopted the absolute value of 3. It is worth mentioning that patients with a BASDAI 2.8 to <4 seem to have significant benefit of anti-TNF therapy. Patient Acceptable Symptomatic State—the highest level of symptoms beyond which patients consider themselves well—after 3 months was rated in a dedicated study at 3.45 [39]. In our study, statistically significant relationships were found for four polymorphisms: *ERAP1* rs2287987, *ERAP2* rs2549782, *TNFRSF1B* rs1061622, and *FCGR2A* rs1801274. Statistically significant data are presented in Table 5.

Associations between ESR improvement of more than 50% were observed within *ERAP1*, *ERAP2*, and *TNFRSF1B* gene polymorphisms. *ERAP1* rs2287987 *AA* homozygotes were more likely to achieve improvement at six months (*AA* vs. *AG + GG*, *p* = 0.047, OR = 2.58, 95% CI 1.098–6.26; *AA* vs. *AG*, *p* = 0.026, OR = 2.751, 95% CI 1.11–6.31), whereas *ERAP2* rs2549782 *T* allele carriers were more frequently observed to show improvement at three months (*GT + TT* vs. *GG*, *p* = 0.037, OR = 2.76, 95% CI 1.13–6.72, *GT* vs. *GG*, *p* = 0.044, OR = 3.04, 95% CI 1.09–8.45). The *TNFRSF1B* rs1061622 *G* variant was associated with ESR improvement at six months (*TG + GG* vs. *TT*, *p* = 0.020, OR = 3, 95% CI 1.27–6.9; *TG* vs. *TT*, *p* = 0.025, OR = 3.16, 95% CI 1.18–7.88). Additionally, for the *TNFRSF1B* rs1061622 polymorphism, an association with the clinical scale BASDAI was noted. Heterozygotes were more commonly characterized by low BASDAI scores (<3) (*TG* vs. *GG*, *p* = 0.047, OR = 8.87, 95% CI 1.21–104) and better treatment response (*TG* vs. *TT + GG*, *p* = 0.045, OR = 2.46, 95% CI 1.00–6.11).

For treatment effectiveness, the highest number of statistical relationships concerns *FCGR2A* rs1801274. This polymorphism may be a marker of the long-term response to anti-TNF treatment, as measured by the BASDAI. *GG* homozygotes were less frequently observed to have BASDAI scores lower than 2 (*GG* vs. *AG*, *p* = 0.046, OR = 0.25, 95% CI 0.070–0.979). Similarly, the *GG* genotype was less commonly identified with low disease activity, defined as a BASDAI score < 3 (*GG* vs. *AA + AG*, *p* = 0.028, OR = 0.22, 95% CI 0.062–0.748; *GG* vs. *AG*, *p* = 0.032, OR = 0.19, 95% CI 0.053–0.833). In addition, the same genotype was less common among patients with a BASDAI improvement of more than 50% (*GG* vs. *AA + AG*, *p* = 0.012, OR = 0.161, 95% CI 0.489–0.594; *GG* vs. *AG*, *p* = 0.007, OR = 0.092, 95% CI 0.017–0.557).

## 4. Discussion

There are > 13 known AS-associated SNPs that span the *ERAP1* gene locus, including rs3734016, rs26653, rs27895, rs2287987, rs27434, rs30187, rs10050860, rs17482078, rs27044, rs1065407, rs27980, rs7711564, and rs27037, which have been used as genetic markers in multiple association studies. A total of eight SNPs (rs3734016, rs26653, rs27895, rs2287987, rs30187, rs10050860, rs17482078, and rs27044) are nonsynonymous substitutions in the coding region of the ERAP1 gene, which implies that the corresponding amino acid substitutions exhibit functional effects [40]. Ethnic differences are important, as some relationships are limited to Caucasians or Asians [21,41]. Significant differences in the minor allele and genotype distribution between patients and controls were found in the Polish population for *ERAP1* rs2287987, rs30187, and rs27044. These results were consistent with those in other European populations, whereas an association of rs2287987 was not observed in Portuguese and Koreans. In contrast to *ERAP1*, no effect of the *ERAP2* rs2248374 SNP was observed in Polish patients with AS. However, *ERAP1-ERAP2* haplotype analysis demonstrated a possible association of both genes with AS. Interestingly, unlike *ERAP1*, *ERAP2* seems to play a role in both HLA-B27-positive and HLA-B27-negative AS patients [18]. The restriction of the association of *ERAP1* with ankylosing spondylitis to HLA-B27-positive patients is consistent with disease models in which aberrant trimming of peptides or presentation by *ERAP1* and HLA-B27 are involved in the pathogenesis of HLA-B27-associated disease. HLA-B27- and ERAP1-negative disease is unlikely to be caused by a similar mechanism, and the fact that the overexpression of TNF alone is sufficient to cause spondyloarthritis in mice suggests that overexpression or signaling by proinflammatory cytokines alone may be sufficient to cause ankylosing spondylitis [42].

Markedly fewer studies have focused on the *ERAP2* gene, and the data are conflicting. The meta-analysis did not support any evidence on the associations for rs2248374 and rs2549782 polymorphisms in the *ERAP2* gene and susceptibility to AS. The authors indicated the need for further studies in large-sample populations and in diverse ethnicities [43]. The interactions between genes are more complex. Interesting are the results of haplotype research. *ERAP2* haplotypes rs2910686/rs2248374/rs2549782 [44] and *ERAP1/ERAP2* rs27044/rs30187/rs2549782 [45] were shown to be more frequent in AS patients. We have expanded the *ERAP* studies of Polish population with *ERAP2* rs2549782.

TNF-α plays a vital role in the typical immune response through the regulation of a number of pathways encompassing an immediate inflammatory reaction with significant innate immune involvement as well as cellular activation with subsequent proliferation and programmed cell death or necrosis [46]. The literature suggests that genetically determined high activity of the TNF-α pathway is associated with an increased risk of AS [47]. A meta-analysis suggested that *TNF-α* polymorphisms rs361525, rs1800629, rs1799724, rs1799964, and rs769178 could influence AS susceptibility in the total population. Some results in the subgroups were not consistent with those in the total population [48]. There was no association in the HLA-B27-positive AS group and HLA-B27-positive control group [34,43]. Another meta-analysis consisting of a larger number of studies proposed that the rs1800629 polymorphism is associated with an increased AS risk in Caucasians and that the rs361525 and rs1800630 polymorphisms are linked to an elevated AS susceptibility in Asians [25]. In our study, we observed some trend in rs1800629, but the *p*-value in the general population was above 0.05. Further analysis showed that the *TNF* rs1800629-*GG* genotype was more common in male patients. Apart from our study, we found no other sex-related correlations in the literature. Available data concern mainly patients with AS. Our study group consists of axSpA patients—both AS and nr-SpA patients.

Most of the research on TNF receptor polymorphisms comes from Asia. In AS, *TFRSF1A* rs4149570 [49], rs4149577 [50], rs767455 [26], and *TNFRSF1B* rs1061622 [51] polymorphisms have been demonstrated. In an extended study in a Caucasian population, there were associations with *TNFRSF1A* rs4149576 and rs4149577 [52]. Reveille et al. found *TNFRSF1A* rs1800693 in individuals of European descent [53].

There are a few data on the prevalence of *FCGR2A* SNPs in AS. The occurrence of rs1801274 among Europeans has been confirmed [54]. Considering other autoimmune diseases, associations with Kawasaki disease and ulcerative colitis have also been demonstrated for *FCGR2A* rs1801274 [35].

There are fewer data in the literature about disease activity dependent on genetic polymorphisms. Nossent et al. showed an association between the *ERAP1* rs27044 *C*/rs30187 *T* haplotype and a reduced risk for extraspinal disease and a lower risk of systemic inflammation in HLA-B27-positive patients. However, this was not explained by any association between the ERAP1 haplotype and proinflammatory cytokine levels. Other studies have shown lower syndesmophyte formation for rs30187 and the *C* allele in Taiwan and lower BASFI scores for rs27044 and the *C* allele in Spain [55]. On the other hand, no relationship was found between six *ERAP1* SNPs, namely rs30187, rs27044, rs27434, rs17482078, rs10050860, and rs2287987 and AS activity as measured by sacroiliac joint inflammation on MRI, BASDAI score, ASDAS-CRP, and CRP in the French population [56]. In contrast, we focused on *ERAP1* rs2287987, which affected the initial CRP value among Polish patients, but this relationship was not statistically significant after Bonferroni correction. A relationship for CRP was also described for *ERAP1* rs27044 in Iran [57].

*TNF* rs1800629 has been described to be associated with disease activity in South and Central American populations [58]. In China, this SNP may be a weak indicator reflecting the active state of AS. The haplotype *GACTCG* of five SNPs in *TNF-α* and one in *TNF-β* may indicate both the susceptibility and the activity of AS [59]. We did not confirm these data in Caucasians.

Similar to the study by Xing-Rong et al., we confirmed the relationship between the *TNFRSF1B* rs1061622 polymorphism and disease activity but assessed it by other parameters. In the Chinese study, apart from chest expansion, no significant difference was found in disease duration, BASDAI and BASFI scores, the duration of morning stiffness, the distance from the occiput to the wall, Schober’s test score, the number of swollen joints, or the number of tender joints [26]. In our study, we found initial VAS and BASDAI score associations with rs1061622. Moreover, we expanded the study to include inflammatory parameters, which were affected by *TNFRSF1A* rs767455. In the abovementioned paper, this polymorphism was not assessed due to the small size of the group of patients with one of the genotypes. Another described polymorphism of *TNFRSF1A* is rs1800693, which is associated with the BASDAI score [60].

Research on the *FCGR2A* gene is related to other inflammatory diseases, such as chronic periaortitis [61] or severe pneumonia in A/H1N1 influenza infection [62]. Other described polymorphisms associated with disease activity are located, for example, within genes coding for IL-33 [63], IL-17F, and IL-17RA [64]. A detailed understanding of the pathogenesis of disease, including the functions of genes involved in this process, will allow the development of drugs with new mechanisms of action. Early selection of patients at risk for severe disease, based on genetic markers, will improve axSpA treatment strategies.

Uveitis is the most common, clinically important extra-articular manifestation of AS. The phenotype characteristic for AS (sudden onset, anterior, unilateral, recurrent, more often male) may differ from the phenotype often seen with either psoriatic arthritis or inflammatory bowel disease (insidious onset, anterior and intermediate, bilateral, chronic, and/or more often female) [65]. Genes associated with the risk of developing uveitis include *ERAP1*, *intergenic region 2p15*, *IL23R*, *IL10-IL19*, *IL18R1-IL1R1*, *IL6R*, *KIF21B,* and *EYS*. This means that overlapping but also distinct genetic susceptibility loci for uveitis and AS were demonstrated [66]. Among the *ERAP1* polymorphisms associated with the risk of uveitis are rs2032890, rs27529, rs30187, and rs2287987 (the relationship is greater for the rs2287987/-rs10044354 haplotype) [67,68,69]. In our study, we did not confirm the relationship between *ERAP1* rs2287987 and the occurrence of uveitis but rather with enthesitis. On the other hand, we showed a relationship between *TNFRSF1B* rs1061622 and uveitis. Consistent with the high heritability, a genome-wide polygenic risk score shows strong power in identifying individuals at high risk of either AS with uveitis or AS alone [67].

There are fewer data on the possibility of peripheral arthritis in axSpA. Among the polymorphisms found more frequently in AS patients with arthritis, *JAK2* rs7857730, *IL-23R* rs11209008 and rs10489630, *CYP1B1* rs1056836, *NELL1* rs8176786, *KL* rs564481, *MEFV* rs224204, *IL-2RB* rs743777, and *IL-1A* rs1800587 have been described [70]. Another study showed an association of the *ERAP1* rs27044 *C*/rs30187 *T* haplotype with a lower risk of extraspinal disease, defined as one of the following: peripheral arthritis, psoriasis, uveitis, and IBD [55].

The *FCGR2A* rs1801274 *A* allele is a good candidate for a potential biomarker of anti-TNF treatment response after six months. Using BASDAI score, we confirmed the relationship for disease activity. For autoimmune rheumatic diseases, the data are mainly related to rheumatoid arthritis, and two meta-analyses have been performed showing the impact of *FCGR2A* rs1801274 [71,72]. The high predictive potential of this gene has also been demonstrated by studies with other biologic drugs, such as rituximab [73] and abatacept [74]. The effect of *FCGR2A* rs1801274 on treatment efficacy in SpAs is more difficult to assess due to the inconsistent results of previous studies. A meta-analysis by Lee et al. did not confirm a relationship in patients with spondyloarthropathy, psoriasis, and Crohn’s disease. However, this was confirmed in a single long-term follow-up study with a follow-up time ≥ 6 months. Similarly, in our study group of axSpA patients, we confirmed the relationship only in the case of a longer period after 6 months of treatment. The mechanism of this relationship was explained: the *FCGR2A* rs1801274 polymorphism affects the affinity of the receptor for IgG. Homozygous neutrophils having a histidine at position 131 have a threefold higher phagocytosis rate and sevenfold higher bactericidal activity than homozygous neutrophils with the arginine 131 genotype. The Fc portions of anti-TNFs bind to FCGR on the cell surface, which may affect cellular functional processes, such as phagocytosis, cytokine release, degranulation, and antibody-dependent cell-mediated cytotoxicity. Anti-TNFs share an IgG1 Fc fragment and may neutralize the biologic activity of TNF-α [75]. It may be possible to use genetic variants to determine prognosis and potentially guide treatment decisions. A key step towards their use would be external validation as well as an investigation of their integration with existing clinical prognostic scores [76].

As mentioned above, the effect of *ERAP* genetic variants on AS prevalence has been widely described. It is worth noting the relationship of rs151823 and rs26653 polymorphisms in the *ERAP1* gene with a good response to ustekinumab, an anti-IL-12/23 agent, in psoriatic patients with early onset of the disease [77]. In the present study, we analyzed another *ERAP1* polymorphism, rs2287987, for which *AA* homozygosity was found to be associated with better ESR improvement.

The hypothesis regarding the effect of TNF-axis gene variation on anti-TNF efficacy seems obvious. Studies in this direction have been conducted. A meta-analysis showed that the presence of the *TNF* rs1800629 *G* allele and the rs361525 *G* allele predicted a good response to TNF blockers and that it was dependent on the response criteria used. However, the prediction of the response to etanercept was much more powerful than the prediction of the response to infliximab/adalimumab. These drugs have a different molecular structure. *TNF* rs1799724 could not predict the response in either subgroup [78]. It is legitimate to ask about genes encoding TNF receptors, such as *TNFRSF1A* and *TNFRSF1B*, and whether they also affect treatment efficacy. In both cases, variants with and without a confirmed relationship can be found. The first group includes *TNFRSF1A* rs1800693 and *TNFRSF1B* rs1061622, and the second group includes *TNFRSF1A* rs767455, rs2234649, and rs4149570 [26,49,79]. Of the three TNF-axis genes tested in our study, namely *TNF* rs1800629, *TNFRSF1A* rs767455, and *TNFRSF1B* rs1061622, only *TNFRSF1B* rs1061622 showed predictive potential for treatment response, measured by an ESR reduction in the six-month follow-up, a BASDAI score < 3 in the third month, and BASDAI improvement > 75% in the sixth month.

There has already been a successful attempt to create a model of anti-TNF nonresponse combining genotype and clinical features. It included the following characteristics: female sex, elevated baseline BASFI scores, and the *CHUK* gene rs11591741-*GG* SNP. The identification of predictors of nonresponse could assist in decision making for an alternative therapy in patients with SpA; it would also help to improve the risk/benefit ratio in patients who are candidates for initiating treatment with anti-TNF agents [80]. A model containing several or more genetic polymorphisms could even more precisely predict treatment response.

The main limitation of the study was the small group size. Genetic studies require a large number of patients. For this reason, we counted the correlations for different anti-TNF drugs together. Racial variability must be borne in mind, and results may differ for other populations around the world.

## 5. Conclusions

This is one of the few genetic studies that addresses axSpA in a comprehensive manner. We assessed genetic polymorphisms in terms of disease prevalence, clinical features, activity, extra-articular symptoms presence, and treatment effectiveness in a short and long period of time. SNPs have the potential to predict the disease course. We report genetic polymorphisms associated with high initial axSpA activity; after Bonferroni correction, *TNFRSF1A* rs767455 and *TNFRSF1B* rs1061622 remained statistically significant. The *ERAP1* rs2287987 *GG* genotype was more frequently observed in patients with enthesitis, while the *TNFRSF1B* rs1061622 *GG* genotype was more common in participants with uveitis than the *TT* genotype. Potential in predicting anti-TNF treatment response was demonstrated by *ERAP1* rs2287987, *ERAP2* rs2549782, *TNFRSF1B* rs1061622, and *FCGR2A* rs1801274. A greater improvement in ESR was observed for *ERAP1* rs2287987 *AA* homozygotes, carriers of the *ERAP2* rs2549782 *T* allele, and carriers of the *TNFRSF1B* rs1061622 *G* allele. Moreover, *TNFRSF1B* rs1061622 heterozygotes were more commonly characterized by low BASDAI scores and better treatment responses.

In clinical practice, such biomarkers can be used to identify patients at risk of severe disease to initiate treatment earlier. Predicting the effectiveness of therapy based on genetic testing will allow clinicians to choose the right drug for the patient, thus personalizing treatment. To develop treatment algorithms, more studies on larger groups of patients are needed.

## Figures and Tables

**Table 1:** Clinical characteristics of the patients’ cohort.

Number of Patients (*N*)	106
Age mean in years (±SD)	42.7 (±12.9)
Disease duration mean in years (±SD)	9.29 (±8.49)
Disease onset mean in years (±SD)	32.7 (±10.2)
Sex F/M (%)	28/78 (73.6%)
BMI mean (±SD)	25.5 (±4.59)
HLA-B27 positive patients, %	88%
Form axial/axially peripheral (%)	58 (54.7%)/48 (45.3%)
nr-axSpA/AS (%)	20 (18.9%)/86 (81.1%)
**Extra-articular manifestations**:	*N* (%)
Uveitis	33 (31.1%)
Inflammatory bowel disease	18 (17.0%)
Enthesitis	17 (16.0%)
Psoriasis	6 (5.66%)
Patients with at least one manifestation	53 (50.0%)
Patients with two manifestations or more	17 (16.0%)
**Concomitant treatment at the start of biologic treatment**:	*N* (%)
NSAIDs	73 (69.5%)
MTX	32 (30.2%)
Sulfasalazine/Mesalazine	28 (26.42%)
Corticosteroids	17 (16.0%)
Other	2 (1.89%)
**Anti-TNF drugs**:	*N* (%)
Adalimumab	43 (40.6%)
Etanercept	28 (26.4%)
Certolizumab	24 (22.6%)
Golimumab	9 (8.49%)
Infliximab	2 (1.89%)
**Disease activity**:	
BASDAI before treatment, median (range)	7.45 (4.05–10)
BASDAI after 6 months of treatment, median (range)	2.30 (0–10)
Low activity * after 6 months of treatment, *N* (%)	97 (93.3%)

SD, standard deviation; F, female; M, male; nr-axSpA, non-radiographic axial spondyloarthritis; AS, ankylosing spondylitis; BMI, body mass index; HLA-B27, human leukocyte antigen B27; MTX, methotrexate; NSAIDs, nonsteroidal anti-inflammatory drugs; BASDAI, Bath Ankylosing Spondylitis Disease Activity Index. * defined as BASDAI < 3.

**Table 3:** The distribution of genotypes and alleles in AS patients and the control group.

	Patients *N* = 106		Controls *N* = 110		*p*-Value χ^2^	*p*-Value Fisher	OR	95% CI
***ERAP1*** **rs2287987**								
AA	70	66.0%	71	64.5%	0.799			
AG	33	31.1%	34	30.9%				
GG	3	2.8%	5	4.5%				
A	173	81.6%	176	80.0%		0.715	1.11	(0.687–1.81)
G	39	18.4%	44	20.0%				
***ERAP2*** **rs2549782**								
GG	25	23.6%	29	26.4%	0.849			
GT	50	47.2%	52	47.3%				
TT	31	29.2%	29	26.4%				
G	100	47.2%	110	50.0%		0.565	0.893	(0.615–1.29)
T	112	52.8%	110	50.0%				
***TNFRSF1B*** **rs1061622**								
TT	64	60.4%	68	61.8%	0.264			
TG	34	32.1%	39	35.5%				
GG	8	7.5%	3	2.7%				
T	162	76.4%	175	79.5%		0.486	0.833	(0.522–1.32)
G	50	23.6%	45	20.5%				
***TNF*** **rs1800629**								
GG	86	81.1%	80	72.7%	0.058			
GA	20	18.9%	25	22.7%				
AA	0	0.0%	5	4.5%				
G	192	90.6%	185	84.1%		0.060	1.82	(0.999–3.25)
A	20	9.4%	35	15.9%				
***TNFRSF1A*** **rs767455**								
TT	34	32.1%	30	27.3%	0.728			
TC	47	44.3%	51	46.4%				
CC	25	23.6%	29	26.4%				
T	115	54.2%	111	50.5%		0.442	1.16	(0.803–1.69)
C	97	45.8%	109	49.5%				
***FCGR2A*** **rs1801274**								
AA	38	35.8%	39	35.5%	0.860			
AG	52	49.1%	57	51.8%				
GG	16	15.1%	14	12.7%				
A	128	60.4%	135	61.4%		0.844	0.959	(0.65–1.42)
*G*	84	39.6%	85	38.6%				

OR, odds ratio; CI, confidence interval.

**Table 4:** Statistically significant associations between ERAP1, TNFRSF1B, TNFRSF1A, AND FCGR2A genotypes and clinical parameters.

		*N*	Min	Q1	Median	Q3	Max	IQR	*p* ^a^		*p* ^b^
***TNFRSF1B* rs1061622**											
**VAS at baseline (mm)**	*TT*	58	45	70	80	85	100	15	0.003	*TT* vs. *TG + GG*	0.653
										*TT + TG* vs. *GG*	0.001 *
	*TG*	34	48	70	79	82.3	93	12.3		*TT + GG* vs. *TG*	0.144
										*TT* vs. *TG*	0.451
	*GG*	8	80	84.8	93	95	100	10.3		*TG* vs. *GG*	0.001 *
										*TT* vs. *GG*	0.002 *
**BASDAI at baseline**	*TT*	64	4.05	5.9	7.3	8.2	9.8	2.3	0.015	*TT* vs. *TG + GG*	0.696
										*TT + TG* vs. *GG*	0.005 *
	*TG*	34	3.95	5.84	7.05	7.93	9.2	2.09		*TT + GG* vs. *TG*	0.236
										*TT* vs. *TG*	0.523
	*GG*	8	6.35	7.78	8.6	9.3	10	1.53		*TG* vs. *GG*	0.004 *
										*TT* vs. *GG*	0.009
***TNFRSF1A* rs767455**											
**CRP at baseline (mg/L)**	*TT*	34	0.68	4.25	18.2	40.1	99.8	35.9	0.017	*TT* vs. *TC + CC*	0.009
										*TT + TC* vs. *CC*	0.991
	*TC*	46	0.35	1.69	5.29	14.8	59.7	13.1		*TT + CC* vs. *TC*	0.014
										*TT* vs. *TC*	0.006 *
	*CC*	25	0.76	2.79	7.85	18.7	51.9	15.9		*TC* vs. *CC*	0.231
										*TT* vs. *CC*	0.120

*N*, number of patients in groups; CRP, C-reactive protein; ESR, erythrocyte sedimentation rate; VAS, Visual Analogue Scale; BASDAI, Bath Ankylosing Spondylitis Disease Activity Index; Q1, lower quartile; Q3, upper quartile; IQR, interquartile range. ^a^ Kruskal–Wallis test. ^b^ Mann–Whitney test, *p* < 0.008 was considered significant (according to Bonferroni correction) and marked by asterisk (*).

**Table 5:** Statistically significant relationships in the assessment of anti-TNF treatment effectiveness.

		Genetic Variants		
***ERAP1*** **rs2287987**		AA	AG	GG
ESR improvement at 6 months > 50%	(+)	49 (72.06%) ^(a)^	15 (48.39%)	2 (66.67%)
	(−)	19 (27.94%)	16 (51.61%)	1 (33.33%)
***ERAP2*** **rs2549782**		GG	GT	TT
ESR improvement at 6 months > 50%	(+)	9 (37.5%)	31 (64.58%) ^(b)^	17 (58.62%)
	(−)	15 (62.5%)	17 (35.42%)	12 (41.38%)
***TNFRSF1B*** **rs1061622**		GG	TG	TT
ESR improvement at 6 months > 50%	(+)	6 (75%)	27 (79.41%) ^(c)^	33 (55%)
	(−)	2 (25%)	7 (20.59%)	27 (45%)
BASDAI 3 months < 3	(+)	1 (12.5%)	19 (55.88%) ^(d)^	31 (48.44%)
	(−)	7 (87.5%)	15 (44.12%)	33 (51.56%)
BASDAI improvement 6 months > 75%	(+)	2 (25%)	15 (44.12%) ^(e)^	15 (24.19%)
	(−)	6 (75%)	19 (55.88%)	47 (75.81%)
***FCGR2A*** **rs1801274**		AA	AG	GG
BASDAI 6 months < 2	(+)	17 (44.74%)	24 (48%)	3 (18.75%) ^(f)^
	(−)	21 (55.26%)	26 (52%)	13 (81.25%)
BASDAI 6 months < 3	(+)	34 (89.47%)	46 (92%)	11 (68.75%) ^(g)^
	(−)	4 (10.53%)	4 (8%)	5 (31.25%)
BASDAI improvement 6 months > 50%	(+)	34 (89.47%)	48 (96%)	11 (68.75%) ^(h)^
	(−)	4 (10.53%)	2 (4%)	5 (31.25%)

(+): the presence of assessed parameter; (−): no parameter assessed; ESR, erythrocyte sedimentation rate; BASDAI, Bath Ankylosing Spondylitis Disease Activity Index; 3 months, evaluation in the third month of anti-TNF treatment; 6 months, evaluation in the sixth month of anti-TNF treatment. ^(a)^ *AA* vs. *AG + GG*, *p* = 0.047, OR = 2.58, 95% CI 1.098–6.26; *AA* vs. *AG*, *p* = 0.026, OR = 2.75, 95% CI 1.11–6.31, *AA + GG* vs. *AG*, *p* = 0.027, OR = 2.72, 95% CI 1.12–6.14; ^(b)^ *GT + TT* vs. *GG*, *p* = 0.037, OR = 2.76, 95% CI 1.13–6.72, *GT* vs. *GG*, *p* = 0.044, OR = 3.04, 95% CI 1.09–8.45; ^(c)^ *TG + GG* vs. *TT*, *p* = 0.020, OR = 3, 95% CI 1.27–6.9; *TG* vs. *TT*, *p* = 0.025, OR = 3.16, 95% CI 1.18–7.88; *TG* vs. *TT + GG*, *p* = 0.030, OR = 2.87, 95% CI 1.11–7.16; ^(d)^ *TG* vs. *GG*, *p* = 0.047, OR = 8.87, 95% CI 1.21–104; ^(e)^ *TG* vs. *TT + GG*, *p* = 0.045, OR = 2.46, 95% CI 1.00–6.11; ^(f)^ *GG* vs. *AG*, *p* = 0.046, OR = 0.25, 95% CI 0.070–0.979; ^(g)^ *GG* vs. *AA + AG*, *p* = 0.028, OR = 0.22, 95% CI 0.062–0.748; *GG* vs. *AG*, *p* = 0.032, OR = 0.19, 95% CI 0.053–0.833; ^(h)^ *GG* vs. *AA + AG*, *p* = 0.012, OR = 0.161, 95% CI 0.489–0.594; *GG* vs. *AG*, *p* = 0.007, OR = 0.092, 95% CI 0.017–0.557.

## Data Availability

The data used to support the findings of this study are available from the corresponding author upon request.

## Supplementary Materials

The following supporting information can be downloaded at: https://www.mdpi.com/article/10.3390/jcm11102912/s1, Table S1. Associations between ERAP1, TNFRSF1B, TNFRSF1A, FCGR2A genotypes and clinical parameters.
