# Supplementary material for: Searching for New Genetic Biomarkers of Axial Spondyloarthritis
Source: J Clin Med. 2022 May 20;11(10):2912. doi: 10.3390/jcm11102912 (PMC9148009; doi:10.3390/jcm11102912)
Supplement: Supplementary file 1 [file jcm-11-02912-s001.zip › jcm-1696747-supplementary.pdf]

Table S1. Associations between ERAP1, TNFRSF1B, TNFRSF1A, FCGR2A genotypes and clinical parameters.

		N	Min	Q1	Median	Q3	Max	IQR	P^a		P^b
				ERAP1 **rs2287987**							
**CRP at baseline [mg/l]**	*AA*	69	0.42	4.29	10.8	26.8	99.8	22.5	0.032	*AA* vs *AG* + *GG*	0.012
										AA + *AG* vs *GG*	0.916
	AG	33	0.35	1.19	2.77	19.2	68.8	18.0		*AA* + *GG vs AG*	0.009
										AA vs *AG*	0.010
	GG	3	3.64	3.64	4.83	37.93	37.9	34.3		*AG* vs *GG*	0.319
										AA vs *GG*	0.735
**CRP at 12th week [mg/l]**	*AA*	69	0.13	0.66	1.65	5.66	26.7	5.0	0.020	*AA* vs *AG* + *GG*	0.066
										AA + *AG* vs *GG*	-
	AG	33	0.1	0.33	0.78	2.075	108.3	1.75		*AA* + *GG vs AG*	0.018
										AA vs *AG*	0.025
	GG	2	5.75	5.75	10.2	14.7	14.7	8.94		*AG* vs *GG*	-
										AA vs *GG*	-
**CRP at 24th week [mg/l]**	*AA*	68	0.08	0.578	1.83	5.73	37.6	5.15	0.015	*AA* vs *AG* + *GG*	0.043
										AA + *AG* vs *GG*	0.134
	AG	32	0.13	0.335	0.635	2.77	27.5	2.43		*AA* + *GG vs AG*	0.009
										AA vs *AG*	0.014
	GG	3	2.77	2.77	4.6	10.2	10.2	7.47		*AG* vs *GG*	0.048
										AA vs *GG*	0.224
				TNFRSF1B **rs1061622**							
**VAS at baseline [mm]**	*TT*	58	45	70	80	85	100	15	0.003	*TT vs TG* + *GG*	0.653
										TT + *TG vs GG*	**0.001***
	TG	34	48	70	79	82.3	93	12.3		*TT* + *GG vs TG*	0.144
										TT vs TG	0.451
	GG	8	80	84.8	93	95	100	10.3		*TG vs GG*	**0.001***
										TT vs GG	**0.002***
**BASDAI at baseline**	*TT*	64	4.05	5.9	7.3	8.2	9.8	2.3	0.015	*TT vs TG* + *GG*	0.696
										TT + *TG vs GG*	**0.005***
	TG	34	3.95	5.84	7.05	7.93	9.2	2.09		*TT* + *GG vs TG*	0.236
										TT vs TG	0.523
	GG	8	6.35	7.78	8.6	9.3	10	1.53		*TG vs GG*	**0.004***
										TT vs GG	0.009
				TNFRSF1A **rs767455**							
**CRP at baseline [mg/l]**	*TT*	34	0.68	4.25	18.2	40.1	99.8	35.9	0.017	*TT vs TC* + *CC*	0.009
										TT + *TC vs CC*	0.991
	TC	46	0.35	1.69	5.29	14.8	59.7	13.1		*TT* + *CC vs TC*	0.014
										TT vs TC	**0.006***
	CC	25	0.76	2.79	7.85	18.7	51.9	15.9		*TC vs CC*	0.231
										TT vs CC	0.120
**ESR at baseline [mm/h]**	*TT*	33	1	13	32	50	93	37	0.027	*TT vs TC* + *CC*	0.013
										TT + *TC vs CC*	0.991
	TC	46	2	4.75	15.5	32.3	70	27.5		*TT* + *CC vs TC*	0.020
										TT vs TC	0.010
	CC	25	2	8	22	38.5	64	30.5		*TC vs CC*	0.257
										TT vs CC	0.132
				FCGR2A **rs1801274**							
**ESR at 24th week [mm/h]**	*AA*	38	1	3	9.5	16.8	57	13.8	0.045	*AA vs AG* + *GG*	0.064
										AA + *AG vs GG*	0.030
	AG	50	1	3	5	12.8	46	9.75		*AA* + *GG vs AG*	0.829

								AA vs AG	0.230
GG	16	1	2	3	6.75	45	4.75	*AG vs GG*	0.103
								AA vs GG	0.013

N: number of patients in groups; CRP: C-reactive protein; ESR: Erythrocyte Sedimentation Rate; VAS: Visual Analogue Scale; BASDAI: Bath Ankylosing Spondylitis Disease Activity Index; Q1: lower quartile; Q3: upper quartile; IQR: interquartile range

[a] Kruskal-Wallis test

[b] Mann-Whitney test, $p < 0.008$ was considered significant (according to Bonferroni correction) and marked by asterisk (*)
